# Biotechnological Phytocomplex of *Zanthoxylum piperitum* (L.) DC. Enhances Collagen Biosynthesis In Vitro and Improves Skin Elasticity In Vivo

**DOI:** 10.3390/pharmaceutics17010138

**Published:** 2025-01-20

**Authors:** Giovanna Rigillo, Giovanna Pressi, Oriana Bertaiola, Chiara Guarnerio, Matilde Merlin, Roberto Zambonin, Stefano Pandolfo, Angela Golosio, Francesca Masin, Fabio Tascedda, Marco Biagi, Giulia Baini

**Affiliations:** 1 Department of Biomedical, Metabolic and Neural Science, University of Modena and Reggio Emilia, 41125 Modena, MO, Italy; giovanna.rigillo@unimore.it; 2 Aethera Biotech s.r.l., 36043 Camisano Vicentino, VI, Italy; giovannapressi@aetherabiotech.it (G.P.); orianabertaiola@aetherabiotech.it (O.B.); chiaraguarnerio@aetherabiotech.it (C.G.); matildemerlin@aetherabiotech.it (M.M.); 3 Eurochem Ricerche s.r.l., 35035 Mestrino, PD, Italy; roberto@eurochemricerche.it (R.Z.); stefano@eurochemricerche.it (S.P.); 4 Keminova Italiana S.B s.r.l., 25060 Cellatica, BS, Italy; angela@keminova.com (A.G.); francesca@keminova.com (F.M.); 5 Department of Life Sciences, University of Modena and Reggio Emilia, 41121 Modena, MO, Italy; fabio.tascedda@unimore.it; 6 Consorzio Interuniversitario Biotecnologie (CIB), 34148 Trieste, TS, Italy; 7 Department of Food and Pharmaceutical Sciences, University of Parma, 43121 Parma, PR, Italy; 8 Department of Physical Sciences, Earth and Environment, University of Siena, 53100 Siena, SI, Italy; giulia.baini2@unisi.it

**Keywords:** *Zanthoxylum piperitum*, lignans, phytocomplex, collagenase, lysyl oxidase, skin

## Abstract

**Background:** *Zanthoxylum piperitum* (L.) DC., commonly known as Japanese pepper, is a deciduous shrub native to East Asia. Its berries are widely used as a spice, known for imparting a distinctive, tingly numbing sensation. Biologically, *Z. piperitum* has antimicrobial, antioxidant, and anti-inflammatory properties, and it is studied for its potential benefits in pain relief and digestive health. This study proposed a novel biotechnological *Z. piperitum* phytocomplex (ZPP) obtained by plant cell culture for skin health, specifically targeting collagen synthesis, extracellular matrix stability, and resilience against cellular stress. Given the bioactivity of *Z. piperitum*, we aimed to analyze its efficacy as a sustainable alternative for skin-supportive applications in cosmetics and supplements. **Methods:** ZPP was produced through stable plant cell cultures, yielding a lignan-rich (3.02% *w*/*w*) phytocomplex. Human fibroblasts (HFFs) were treated with varying ZPP concentrations to assess cellular viability, collagen metabolism, and ECM-related enzyme activities, both under normal and cell stress conditions. The in vivo assessment was performed by measuring biophysical skin parameters such as hydration, elasticity, and roughness in female volunteers for a period of six weeks. **Results:** In vitro, ZPP exhibited non-cytotoxicity at all concentrations tested. Under hyperosmotic stress, ZPP reduced cellular damage, suggesting enhanced resilience. ZPP upregulated lysyl oxidase (LOX) protein levels, critical for collagen cross-linking and ECM stability, with protective effects observed under oxidative/inflammatory conditions. Additionally, ZPP selectively inhibited collagenase, attenuating collagen breakdown, though antioxidant activity was modest. In vivo evaluation highlighted improved skin hydration, elasticity, and roughness. **Conclusions**: ZPP shows promise as a biotechnological agent for skin health, particularly in supporting collagen integrity, ECM stabilization, and cellular resilience under stress. While further studies are needed to explore its full efficacy, especially for aging and environmentally stressed skin, these findings highlight ZPP’s potential as a new ingredient for cosmetic formulations aimed at skin care and the treatment of alterations caused by aging or environmental conditions.

## 1. Introduction

*Zanthoxylum piperitum* (L.) DC., commonly referred to as Japanese pepper or Japanese prickly ash, is a deciduous shrub of the Rutaceae family. Native to East Asia, this species has been cultivated and utilized for centuries across Japan, Korea, and China for its unique culinary, medicinal, and cosmetic properties [1,2]. The small, aromatic drupes produced by *Z. piperitum* are renowned for their distinctive numbing and tingling sensation, coupled with a citrusy flavor, making them a staple in various Asian cuisines.

The multifaceted applications of *Z. piperitum* extend far beyond its culinary uses. Traditional Asian medicine has long valued this plant for its analgesic, anti-inflammatory, digestive, and detoxifying properties [3]. Recent phytochemical studies have identified several bioactive compounds within *Z. piperitum*, such as alkylamides (including sanshool and hydroxy-α-sanshool), which are primarily responsible for its therapeutic effects. These compounds have shown potential in providing pain relief, anti-allergic effects, reducing inflammation, and enhancing digestive health [4,5,6].

Preclinical studies have also highlighted the pharmacological benefits of the plant’s volatile compounds, such as limonene which has shown significant anti-inflammatory, antioxidant, anti-bacterial, and anti-proliferative effects [7,8,9,10,11]. Other compounds like citronellal and geranyl acetate also demonstrate biological regulatory functions. Notably, lignans presence in the phenolic fraction of *Z. piperitum* stems showed potential antioxidant and anti-osteoporosis activities [12].

Despite its extensive traditional use and emerging applications, *Z. piperitum* remains scarcely explored in Western medicine. This presents a substantial opportunity for further scientific investigation to uncover its full potential and integrate its benefits into modern therapeutics as well as in nutraceutics or cosmetic practice. Indeed, *Z. piperitum* has recently garnered significant attention in aging-related skin modifications for its skin-soothing, anti-wrinkle benefits and photoprotective effects [13,14,15]. These studies also explored the role of different constituents of *Z. piperitum*: alkylamides, such as α-sanshool and hydroxy-α-sanshool, have been demostrated to exert a strong anti-inflammatory activity in lypopolysaccharide-stimulated macrophages (RAW 264.7) [13]; α-sanshool has been shown to protect human fibroblasts against damages produced by UVB irradiation by inhibiting the activation of JAK2-STAT3 signaling that hinders the upregulation of metalloproteases, cell viability, and autophagy [14]. On the other hand, in the work of Hwang et al. (2020) [15], *Z. piperitum* fruit extract showed botulinum toxin type A (BoNT/A)-like activity in an In vitro screening assay of BoNT/A-like muscle contraction inhibitors; in particular, quercetin-3-*O*-rhamnoside (quercitrin) seemed to play a major role. Hence, alkilamides and flavonoids have been reported to mainly contribute to the biological effects of *Z. piperitum* for skin protection, but the role of other compounds such as lignans as well as of the whole phytocomplex still remains unexplored.

Aging is a complex and multifactorial process accompanied by several functional and aesthetic changes on the skin, closely related to reducing elastin and collagen fibers, leading to fine lines and wrinkles [16,17,18,19]. The metabolism of collagen is ensured by a balanced process of synthesis, maturation, and degradation of collagen proteins that begins in the fibroblasts, continues in the extracellular matrix (ECM) and ends outside the cell, where procollagen is cleaved by specific enzymes to form mature collagen. Collagen degradation is regulated by matrix metalloproteinases (MMPs) which break down collagen fibers into smaller fragments [20]. The balance between collagen synthesis and degradation is crucial for maintaining tissue homeostasis and is influenced by various factors, including growth factors, cytokines, and mechanical stress [21]. Over time, reduced collagen production, primarily type I collagen produced by fibroblasts, decreases dermal thickness and contributes to skin wrinkling. Anti-aging strategies often target fibroblast activity to boost ECM production, using ingredients like bioactive peptides, antioxidants, retinoids, vitamins, fatty acids, growth factors, hydroxy acids, and botanical extracts to influence collagen production [19,22,23]. 

Due to the recent surge in the global demand for active plant ingredients in the pharmaceutical and cosmetic market, medicinal plant species are now facing severe mass exploitation, which may ultimately lead to their extinction. In vitro plant cell culture technology offers a solution by providing a sustainable supply of active biomolecules without over-exploiting natural plant biomass [24,25]. This innovative technology allows us to grow plant cells under strictly controlled environmental conditions. Additionally, using plant cell culture instead of cultivated plants for active biomolecule production can overcome limitations related to inconsistent quality caused by seasonal changes, cultivation methods, and geographic variations. Thanks to the high level of process control, it is possible to address the challenges associated with the variability of plant extracts. This approach enables the production of preparations with a reproducible and standardized active substance content. Phytocomplexes obtained through plant cell culture technology can be easily standardized, ensuring they meet the safety requirements for contamination-free and phytochemically consistent products, avoiding batch-to-batch inconsistencies. Traditional plant extracts often exhibit significant variability in composition, influenced by factors such as climate, soil conditions, and cultivation techniques. This variability makes it difficult to ensure the consistent efficacy of extracts in health care applications. Moreover, this controlled technology can significantly enhance the production of active biomolecules through the elicitation of stress (both biotic and abiotic) conditions. In recent years, an innovative biotech platform based on in vitro plant cultures has been developed, using an eco-designed and certified approach named CROP^®^ (Controlled Release of Optimized Plants) (Patent: EP3942017A1, US20220175654A1, EP3941188A1, US20220339232A1) [26]. This non-GMO technology utilizes cellular totipotency to replicate plants from simple tissue fragments, producing a highly standardized and safe phytocomplex. CROP^®^ addresses the challenge of sourcing rare active ingredients, bypassing geographical and seasonal limitations. The platform boasts one of the largest production capacities in Europe and ensures quality and traceability by integrating the entire supply chain within a single automated facility. We recently investigated the safety and biological activity of some CROP^®^-based biotechnological phytocomplexes, such as *Rosa chinensis* Jacq [27], *Perilla frutescens* (L.) Britton [28,29] and *Rhus coriaria* L. [30]. 

Recently, we developed a novel and standardized biotechnological *Z. piperitum* phytocomplex (ZPP) which was alkylamides-free with a high lignan content by utilizing in vitro plant cell culture. This phytocomplex is free from pesticides, contaminants, and residual solvents while maintaining consistent biological efficacy across all batches. In this study, we aimed to propose the novel ZPP as a new cosmetic ingredient for the prevention or handling of aging-damaged skin. The safety and effectiveness of the new biotechnological ZPP were investigated first in different in vitro models on human fibroblast cells in which the cytotoxicity, biological activity, and molecular mechanisms were explored. Once the safety and the biological activity were established, an in vivo study was conducted to assess the efficacy of the ZPP formulation, previously verified for skin tolerance and compatibility, by testing the topical application of a ZPP-based cream in women volunteers.

## 2. Materials and Methods

### 2.1. Zanthoxylum piperitum Cell Suspension Culture

For the cell line induction, the *Zanthoxyllum piperitum* (L.) DC. plant was bought from the nursery “Le Georgiche” (Brescia, Italy). The botanical origin of *Z. piperitum* was confirmed by fingerprint analysis from Parco Tecnologico Padano, Lodi, Italy [31]. Young aerial parts from the *Z. piperitum* plant were collected for the callus induction. The young aerial parts were washed under running water and sterilized by means of a treatment in sequence with 70% (*v*/*v*) ethanol (Honeywell, Seelze, Germany) in water for about 1 min. They were then washed in 2% (*v*/*v*) sodium hypochlorite solution (6–14% active chlorine, Merck KGaA, Darmstadt, Germany) and 0.1% (*v*/*v*) Tween 20 (Duchefa, Haarlem, The Netherlands) for about 3 min; finally, they were given at least 4 washes with sterile distilled water. The aerial parts were cut into small pieces (explants) of sub-centimetric dimensions (0.1–0.5 cm). The fragments of plant tissue were deposited in several Petri dishes containing solid Gamborg B5 Medium [32] supplemented with 20 g/L sucrose (Sudzucker AG, Manheim, Germany), 1 mg/L of naphthalenacetic acid (NAA) (Duchefa), 0.2 mg/L of indolacetic acid (IAA) (Duchefa), 1 mg/L of Kinetin (K) (Duchefa), 1 g/L of Soy Peptone (Duchefa), and 0.8% *w*/*v* of plant agar (Duchefa), final pH 6.5 (*Z. piperitum*-medium, herein called ZP-medium).

Petri dishes containing explants were incubated at 25 ± 2 °C in the dark. Calli were grown after 4 weeks of incubation and were subjected to subculture for at least 8 months until they became friable and homogeneous, with a constant growth rate. The suspension cultures were obtained by transferring a part of selected calli (10% *w*/*v*) into 1 L Erlenmeyer flasks containing 250 mL of liquid culture medium (ZP-medium without plant agar). The suspension cultures were incubated at 25 ± 2 °C and placed in the dark on a rotary shaker at 120 rpm for at least 7 days to reach a fresh weight between 40 and 50% *v*/*v*. The volume of suspension culture was increased to 3 L flasks containing 1 L of fresh ZP liquid culture medium. The amount of suspension culture inoculated into the liquid culture medium was equal to 12% *v*/*v*. To increase the content of lignans, after 7 days of growth the suspension culture was inoculated in a bioreactor of 5 L volume containing 3 L of liquid medium with a different quali-quantitative composition with respect to the ZP liquid medium. Several combinations of salts, nutrients, and plant growth hormones were tested, and the composition that allowed us to maximize the content of the total lignans was Gamborg B5 with the addition of 40 g/L of sucrose, 1 mg/L of NAA, 0.2 mg/L of IAA, 1 mg/L of K, and 1 g/L of Soy Peptone, final pH 6.5 (ZP final liquid medium). After 7 days of growth in ZP final liquid medium, 7.5 mg/L of methyl jasmonate (Merck KGaA was added to induce a higher total content of lignans. Methyl jasmonate (MeJa) solution was prepared by dissolving 400 mg of MeJa in 10 mL of 70% (*v*/*v*) ethanol–water solution. The final suspension culture was grown in ZP final liquid medium for a culture cycle of 14 days, reaching a fresh weight between 75 and 80% *v*/*v*.

### 2.2. Phytocomplex Preparation from Zanthoxylum piperitum Selected Cell Line

After 14 days of growth in a 5 L bioreactor, at 25 °C and in the dark, the *Z. piperitum* cell culture suspensions were filtered by a 200 µm mesh filter and the liquid culture medium was discarded. Cells were washed with a double volume of saline solution (0.9% *w*/*v* NaCl in sterile water) and then homogenized with ultraturrax at 15,000 rpm for 20 min. The biomass of cells was dried to obtain a phytocomplex (ZPP). Drying of the homogenate cells was performed using a Mini Spray Dryer (BUCHI-B290, BÜCHI Labortechnik AG, Flawil, Switzerland). The drying process was carried out by setting the following parameters in the Spray Dryer: 180 °C = inlet air temperature; 12% = rate of pump sample output; 55 mm = gas volume; and 103–104 °C = outlet air temperature.

### 2.3. UPLC-DAD Analysis

UPLC-DAD analysis was used for the quantification of the total lignans in the *Z. piperitum* phytocomplex (ZPP). ZPP powder (100 mg) was dissolved in EtOH 30% *v*/*v*. The suspension was mixed for 30 s with a vortex mixer and sonicated for 15 min in an ice bath; finally, it was centrifuged at 4000 rpm for 15 min at 6 °C. At the end of centrifugation, the supernatant was recovered, diluted 10-fold, filtered to 0.22 µm, and analyzed. Five independent replicates of the ZPP were extracted and measured. The chromatography system used for the quantification of total lignans consisted of an Acquity UPLC BEH C18 1.7 µm column, size 2.1 mm × 100 mm, coupled to an Acquity UPLC BEH C18 1.7 µm VanGuard Pre-column 3/Pk, size 2.1 mm × 5 mm. The platform used for the UPLC-DAD (Ultra Performance Liquid Chromatography-Diode Array Detection) analysis comprised a UPLC system (Waters Corporation, Milford, MA, USA) consisting of an eluent management module, Binary Solvent Manager model I Class, and an auto-sampler, Sample Manager-FTN model I Class, coupled to a PDA (Photo Diode Array) eλ diode array detector. The data were acquired and analyzed using Empower 3 (Waters) software, version Feature Release 4. The chromatography method used was the following: Solvent A: water, 0.1% formic acid; solvent B: 100% acetonitrile. The initial condition was 99% solvent A; moreover, the flow remained constant at 0.350 mL/min throughout the duration of the analysis. The chromatography column was temperature controlled at 30 °C. For the quantification of the total lignans, the chromatogram obtained at the wavelength of 270 nm was used. The total lignans were quantified with the calibration curve of the authentic commercial standard of pinoresinol (purity ≥ 95.0%, #40674, Merck KGaA). The data analysis was carried out with Empower 3 software.

### 2.4. Cell Culture and Treatment

The human foreskin fibroblasts (HFFs) were cultured at 37 °C and 5% CO_2_ in DMEM (Merck KGaA) supplemented with 10% heat-inactivated fetal bovine serum (FBS) (Merck KGaA), 1% penicillin/streptomycin solution (Merck KGaA), and 1% L-glutamine (Merck KGaA), and passed by trypsinization. ZPP stock solution was prepared by solubilizing the extract in the related cell culture medium. The osmotic stress condition was induced by 500 mOsm of NaCl for 8 h. The inflammatory and oxidative stress conditions were induced by 500 ng/mL of bacterial lipopolysaccharide (LPS from Gram-negative *Escherichia coli*, #L3129, Merck KGaA) in association with 300 nM of H_2_O_2_ for 6 h. The immunosuppressive stimulus was induced by 100 µg/mL of methylprednisolone (MetPRED) for 6 h. The HFFs were pre-treated with different concentrations of ZPP (1, 10, and 100 µg/mL in DMEM medium) for 2 h before the stress stimuli. The control group received the related fresh or unconditioned cell medium. Cells were collected at time points indicated in each section for further analysis.

### 2.5. Cell Viability

Cell viability was tested using the Cell Counting Kit-8 (CCK-8, Merck KGaA) as previously described [33,34]. Briefly, HFF cells (5 × 10^4^) were cultured in 96-well plates. After 24 h, cells were treated with ZPP at 1, 10, and 100 µg/mL, respectively, for 24 h, as previously described [29,30], then CCK-8 solution was added to each well and incubated at 37 °C, 5% CO_2_ for 60 min. Cell viability was calculated by measuring the absorbance at 450 nm. Two independent experiments in triplicate were performed (n = 6).

### 2.6. Pro-Collagen I, Elastin, Metalloproteinase 1 (MMP-1), and Metallopeptidase Inhibitor 1 (TIMP-1) Dosage

HFF cells (5 × 10^3^) were seeded into 96-well plates and grown for 24 h. Cells were treated with ZPP 100 µg/mL in DMEM 1% FBS for 24 h; then, supernatants were collected. Pro-collagen I, elastin, MMP-1, and TIMP-1 dosage were measured by using non-competitive sandwich ELISA (Human Pro-Collagen I α-1 Simple-Step kit ab210966, Abcam, Cambridge, UK; Human Elastin ELISA ab239433, Abcam; Human MMP-1 BMS2016-2, Afflimetrix-Thermofisher, Santa Clara, CA, USA; and Human TIMP-1 MA5-13688 (Afflimetrix-Thermofisher) according to the manufacturer’s instructions. Positive controls furnished by Abcam were used. Two independent experiments in triplicate were performed (n = 6).

### 2.7. Western Blot

HFF cells (1 × 10^5^) were seeded in 12-well plates and grown for 24 h. Cells were pre-treated for 2 h with ZPP (1, 10, and 100 µg/mL), then stimulated with LPS (500 ng/mL) and H_2_O_2_ (300 nM) for 6 h or with methylprednisolone (100 µg/mL) for 6 h. After treatment cells were collected and washed twice with PBS 1× then lysed in 1× SDS sample buffer (25 mM Tris–HCl pH 6.8, 1.5 mM EDTA, 20% glycerol, 2% SDS, 5% β-mercaptoethanol, 0.0025% Bromophenol blue). Protein extracts were quantified, and equivalent amounts of extracts were resolved by SDS–polyacrylamide gel electrophoresis (PAGE) and electrotransferred to Amershan^TM^ nitrocellulose membrane (Merck KGaA) [35]. The membrane was first blocked for 2 h with 5% skim milk in 1× TBS and incubated overnight at 4 °C with primary antibodies, rabbit anti-LOX (1:1000, #GTX03201, Genetex, Irvine, CA, USA) and mouse anti-Vinculin (1:5000, V4504, Merck KGaA), in blocking buffer. The following day, the membrane was incubated with anti-rabbit or anti-mouse IgG-HRP-linked antibody (1:5000, Cell Signaling, Danvers, MA, USA), respectively, after washes with TBS-tween 0.1%. Bands were detected using Immobilon Western Chemiluminescent HRP (Millipore, Burlington, MA, USA) and detected by means of an imager instrument (GE Healthcare, Amersham, UK). Quantification of signal optical density was performed by ImageJ Java software version 1.8.0. Two independent experiments in duplicate were performed (n = 4).

### 2.8. Collagenase Activity

The ZPP inhibition activity of collagenase was assessed by using colorimetric cell-free enzymatic “Collagenase” (ab196999, Abcam,) according to the manufacturer’s instructions. Different concentrations of ZPP were tested (100–250–500–1000 µg/mL) in comparison to the positive control provided by the kit (1,10-phenantrolyne 1 M). The tests were performed and optimized in triplicate with their respective controls by calculating the IC_50_ values. The percentage of enzyme inhibition activity exhibited by ZPP was evaluated by comparing the enzymatic activity at different time points, compared to the positive control, and was calculated with the following formula:

$$\frac{\left(\frac{-\Delta A_{test}}{\Delta T}-\frac{-\Delta A_{Reagent\;background}}{\Delta T}\right)}{0.53\times V}\times RV\times DF$$

where

∆*A*345 nm = difference between Abs T2 and Abs T1 at 345 nm

∆*T* = difference between T2 and T1

*DF* = dilution factor

0.53 = millimolar extinction coefficient of collagenase substrate

*V* = enzyme volume (mL)

### 2.9. ORAC (Oxygen Radical Absorbance Capacity) Assay

The antioxidant activity of ZPP was assessed using the ORAC (Oxygen Radical Absorbance Capacity) assay. For this, ZPP was tested at a concentration of 1 mg/mL. The assay was conducted in accordance with the manufacturer’s instructions, utilizing the ORAC Antioxidant Capacity Assay Kit (KF01004, BQC Redox Technologies, Oviedo Asturias, Spain). Fluorescence was measured at an excitation wavelength of 485 nm and an emission wavelength of 528 nm over a period of 30 min, with readings taken at 3 min intervals using the PerkinElmer VICTOR^®^ NivoTM Multimode Microplate Reader. Values were expressed as Trolox Equivalent Antioxidant Capacity (TEAC), calculated based on a calibration curve generated with standard grade Trolox.

### 2.10. In Vivo Test

The cosmetic formulations containing ZPP or placebo used in the in vivo test was produced by the Keminova Italiana S.B s.r.l. Laboratory, (Cellatica, Brescia, Italy) as described in the Supplementary Information.

The safety of the cosmetic formulation was assessed prior to the in vivo efficacy evaluation by means of an occlusive Finn Chamber patch test, performed by the Eurochem Ricerche s.r.l. laboratory (Mestrino, Padua, Italy), certified by ISO 9001:2015 [36], following the guidelines for cosmetic ingredient testing from the Scientific Committee on Consumer Safety (Supplementary Materials). Skin tolerance and compatibility of formulation ingredients were evaluated in order to exclude possible irritant effects according to legal requirements. The product was classified as not irritating if applied to intact human skin (Eurochem report number RAP25711).

The test was conducted in accordance with the Declaration of Helsinki [37], involving 30 female volunteers with an average age of 52 years. Participants were selected based on the following criteria: Caucasian ethnicity; females aged 35 to 65 in general good health; individuals capable of following all study instructions and attending scheduled visits for the study’s duration; participants who provided informed consent; and those who avoided UV exposure and tanning during the study period. The exclusion criteria included the following: pregnant or nursing women; individuals with a history of skin reactions to cosmetic products, detergents, or sensitivity to any product components; those taking topical or systemic medications that could interfere with the results (e.g., anti-inflammatory drugs, corticosteroids); participants with systemic diseases or skin disorders (e.g., eczema, psoriasis, dermatitis); and those who had participated in similar studies within the 30 days prior to this one. During the study, subjects were instructed not to use any products other than those provided for the test area and to avoid UV exposure. The volunteers were divided into two groups: 15 received the active treatment (verum) and 15 received the placebo. The treatment (verum or placebo) involved applying the cream to clean skin on the neck and décolleté twice daily (morning and evening) for 6 weeks, using a sufficient amount to cover the entire area and massaging gently until absorbed. Instrumental skin measurements were taken on the neck and décolleté at the start of the study and after 6 weeks of treatment. These measurements were conducted in a room with controlled temperature and humidity (24 ± 2 °C; 50 ± 10% RH). Volunteers were instructed not to wash their neck and décolleté for at least two hours before assessments and to avoid applying any cosmetics for at least 12 h beforehand. The in vivo tests were conducted by the Eurochem Ricerche s.r.l. laboratory following the guidelines for cosmetic ingredient testing from the Scientific Committee on Consumer Safety [38,39].

#### Instruments and Parameters

The instrumental efficacy of the treatments was tested according to the following skin parameters: deep hydration (measured by the MoistureMeterEpid, Delfin Technologies Ltd., Kuopio, Finland), elasticity (measured by the ElastiMeter, Delfin Technologies Ltd., Kuopio, Finland), and skin roughness (measured by the 3D^®^ Antera system with CS software version 3.1.8, Miravex Limited, Dublin, Ireland). The MoistureMeterEpiD generates a high-frequency electromagnetic (EM) wave (300 MHz) that is transmitted to the skin. The reflected EM wave is recorded, and the resulting dielectric constant is converted by the instrument into a percentage representing the water content in the tissue (PWC%, from 0 to 100). The percentage of water is proportional to skin hydration, with higher values indicating increased water content. The instrument measures hydration up to a depth of 1 mm within the tissue. The effectiveness of the active ingredient in enhancing deep skin hydration compared to the placebo is indicated by a more substantial increase in the PWC% at the end of the treatment. The ElastiMeter measures instantaneous skin elasticity (ISE) without the skin alterations that traditional instruments may cause. It consists of a 0.3 mm indenter, a reference plate, and built-in force sensors. The probe head is briefly pressed against the skin, and the indenter imposes a constant deformation when the reference plate makes full contact with the skin. The skin’s resistance to this deformation is measured as ISE, expressed in N/m. This method is based on the biomechanical response of the skin and subcutaneous tissue, modeled through computational 3D analysis (Finite Element Method). The effectiveness of the active ingredient in improving skin elasticity compared to the placebo is evidenced by a greater increase in the ISE value at the end of treatment. The Antera 3D^®^ system, developed by Miravex Limited, captures high-resolution images using an innovative optical method and mathematical algorithms to generate three-dimensional representations of the skin. This allows for the extraction of data related to the skin’s three-dimensional structure, enabling the evaluation of treatment effectiveness and monitoring of changes over time. The images were analyzed using Antera 3D^®^ CS software version 3.1.8, which employs the “Spot-on” algorithm to automatically align and compare multiple images, compensating for any shifts or rotations between them. The software measures the following parameters:

- Ra: Average roughness, expressed in μm, representing the mean deviation from a straight line, regardless of the vertical direction.
- Rt: Maximum height, expressed in mm, indicating the maximum height difference from peak to trough within the evaluation length.

The anti-wrinkle efficacy was assessed in the décolleté area.

### 2.11. Statistical Analysis

In vitro tests: Data were first analyzed for normality assumption using the Kolmogorov–Smirnov one-sample test for normality: all targets displayed a normal distribution. Differences between groups were analyzed by using one-way analysis of variance (ANOVA) followed by multiple comparison with Dunnett’s or Tukey’s post hoc tests (with *p* < 0.05 significance level), according to experimental design. Data are presented as mean ± S.D. Analyses and graph creation were conducted using Graphpad Prism 8.0 (San Diego, CA, USA). Data were considered statistically significant with a *p*-value < 0.05.

In vivo tests: Data were first analyzed for normality assumption using Shapiro–Wilk tests. As data were non-normal, Friedmann’s test followed by Wilcoxon’s signed rank test for paired data, with Holm’s correction for repeated data, was applied. In all cases, significance was fixed, with a *p*-value < 0.05 considered significant. Only for the comparison of Ra values between the active product and the placebo product at 6 weeks a *p*-value < 0.10 was considered significant. Statistical analysis was processed using the R software version 4.4.2.

## 3. Results

### 3.1. Obtaining a Zanthoxylum piperitum Phytocomplex from a Selected Cell Line and Chemical Analysis

**Figure 1:**
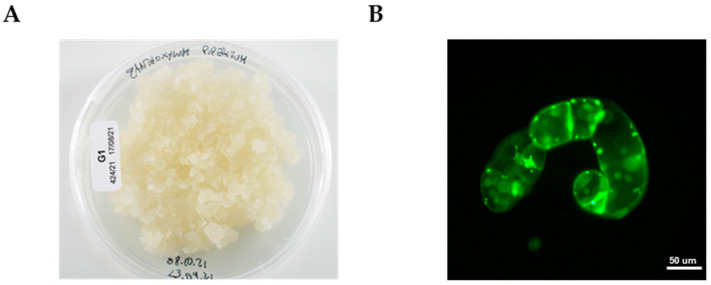
(**A**) *Zanthoxylum piperitum* (L.) DC. cell culture maintained in solid ZP medium. (**B**) Optical images of *Z. piperitum* cells observed by AXIO-Imager A2 optical microscope (ZEISS) after staining with fluorescein diacetate. Scale bar: 50 µM.

**Figure 2:**
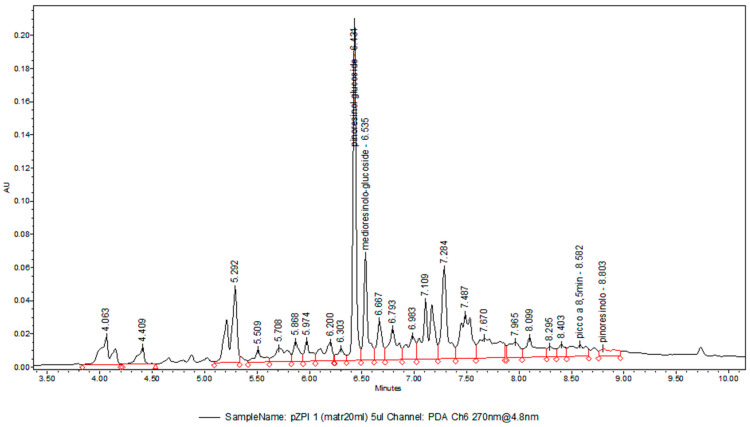
Representative UPLC chromatogram of *Zanthoxylum piperitum* (L.) DC. phytocomplex recorded at 270 nm. Retention time (RT) is indicated at each peak. Pinoresinol glucoside RT = 6.431 min.

A stable cell line of *Z. piperitum* was successfully established using ZP solid medium, which consisted of Gamborg B5 medium supplemented with 20 g/L sucrose, 1 mg/L NAA, 0.2 mg/L IAA, 1 mg/L kinetin (K), 1 g/L soy peptone, and 0.8% (*w*/*v*) plant agar at pH 6.5. After eight months of subculturing on this medium, the *Z. piperitum* cell line developed a brown coloration, a friable texture, and exhibited a high growth rate, requiring transfer to fresh solid medium every 14 days (Figure 1A). Fluorescein diacetate staining was used to assess the morphology and viability of the plant cells maintained on the ZP solid medium (Figure 1B). The total lignan content in the cell line was optimized by transitioning to ZP liquid culture medium, which had an increased sucrose concentration (40 g/L) and was supplemented with 7.5 mg/L MeJa after 7 days of fermentation. Cells grown in the optimized liquid medium were subsequently used to prepare the ZPP. To quantify the total lignan content in the ZPP, UPLC-DAD analysis was conducted. The chromatogram, recorded at 270 nm, is presented in Figure 2. The total lignan content, identified based on their characteristic spectra and expressed as pinoresinol equivalents, was determined to be 3.02 ± 0.15% (*w*/*w*). According to the UV spectra and retention times, we could also exclude the presence of alkylamides if they were not below the limit of quantification.

### 3.2. Effects of ZPP on Cell Viability of Human Fibroblasts

**Figure 3:**
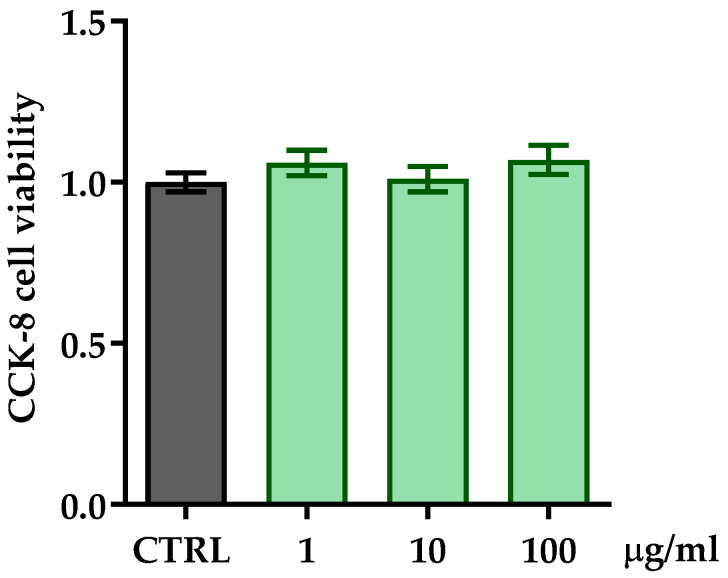
Cell viability analysis by CCK-8 assay on HFF cells treated with ZPP at concentrations of 1, 10, and 100 µg/mL for 24 h. Each column represents mean ± SD. Control cells (CTRL) were arbitrarily set to 1. Data were analyzed by one-way analysis of variance (ANOVA) followed by Dunnett’s post hoc multiple comparison: *p* > 0.05 (n = 6).

The evaluation of the biological activity of ZPP concerned its effects on the viability of fibroblast cells, the main cell type composing the dermis. HFF cells were treated with different concentrations of ZPP (1, 10, 100 µg/mL) for 24 h, then cytotoxicity was investigated via the measurement of cellular metabolism by using the CCK-8 assay. The statistical analysis showed that ZPP did not affect HFF viability at all the concentrations tested compared to untreated cells (one-way ANOVA: F(3,12) = 0.813, *p* = 0.5111) (Figure 3).

### 3.3. ZPP Protects Fibroblasts in an In Vitro Dehydrating Hyperosmotic Stress Model

**Figure 4:**
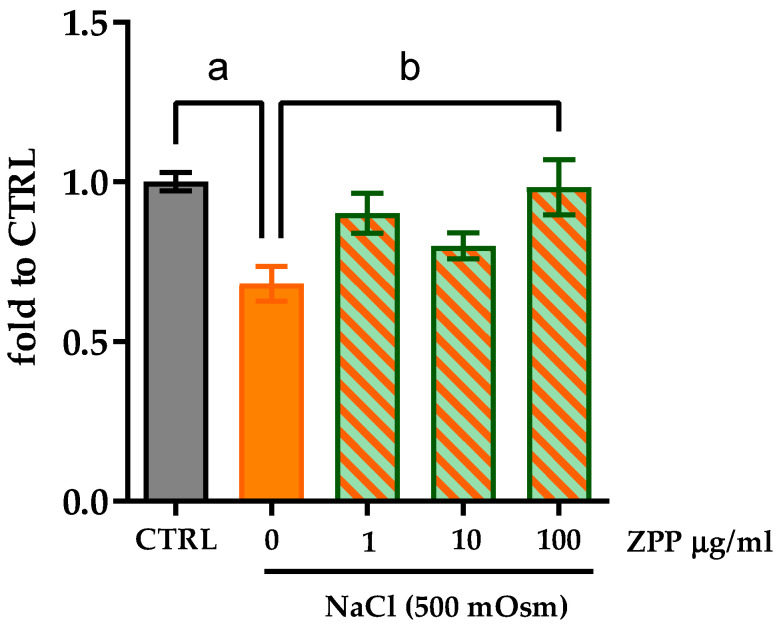
Cell viability analysis by CCK-8 assay on HFF cells pre-treated with ZPP at concentrations of 1, 10, and 100 µg/mL for 2 h then exposed to NaCl (500 mOsm) for 8 h. Each column represents mean ± SD. Control cells (CTRL) were arbitrarily set to 1. Data were analyzed by one-way ANOVA F(4,31) = 5.758; *p* = 0.0014, followed by Tukey’s post hoc multiple comparison: (a) *p* < 0.01 vs. CTRL, (b) *p* < 0.01 vs. NaCl (n = 6).

After ensuring that ZPP had no impact on fibroblast cell viability, we investigated the effect of ZPP on cell viability after an osmotic stress exposure. The effects of pre-treatment with ZPP (1, 10, 100 µg/mL) for 2 h followed by the exposure to NaCl (500 mOsm) for 8 h on HFF cells were measured through the CCK-8 assay. The osmotic stress significantly reduced the cell viability of fibroblasts with respect to the control cells (one-way ANOVA: F(4,31) = 5.758; *p* = 0.0014) (Figure 4), but pre-treatment with ZPP was able to inhibit the NaCl’s impact on cell viability, although we only observed statistical significance (*p* < 0.05 vs. NaCl) at the concentration of 100 µg/mL (Figure 4).

### 3.4. ZPP Does Not Stimulate Elastin Production but Enhances Pro-Collagen I Synthesis

**Figure 5:**
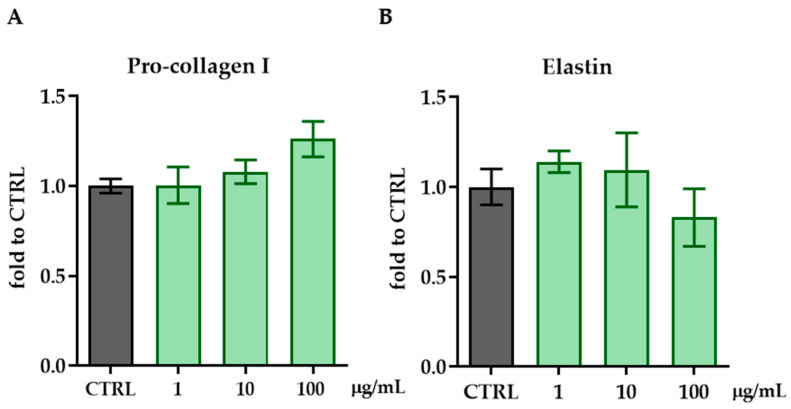
Pro-collagen I (**A**) and elastin (**B**) levels measured by ELISA assay in HFF cells treated with ZPP at concentrations of 1, 10, and 100 µg/mL. Each column represents mean ± SD. Control cells (CTRL) were arbitrarily set to 1. Data were analyzed by one-way ANOVA followed by Dunnett’s post hoc multiple comparison: *p* > 0.05. Pro-collagen I: F(3,13) = 2.046; *p* = 0.1571).Elastin: F(3,4) = 0.927; *p* = 0.5050. (n = 6).

To ensure the maintenance of the physiological state of elasticity and health of the skin, the presence of collagen and elastin is crucial, due to their primary role in providing structure, strength, stretchiness, and support to the skin. For this reason, we evaluated the effects of ZPP, at all the concentrations found to be safe in the previous cytotoxicity tests, in stimulating collagen and elastin synthesis in HFF cells. The levels of pro-collagen I and elastin were measured by ELISA assay after treatment with ZPP (1, 10 and 100 µg/mL) for 24 h. The results showed that ZPP at 100 µg/mL stimulated the synthesis of pro-collagen I with an increase of about 20% compared to untreated cells, despite being non-statistically significant (one-way ANOVA: F(3,13) = 2.046; *p* = 0.1571; Figure 5A). No effects of ZPP in modulating elastin production were observed (Figure 5B).

### 3.5. ZPP Modulates MMP-1 and TIMP-1 Levels Under an Immunosuppressive Condition

**Figure 6:**
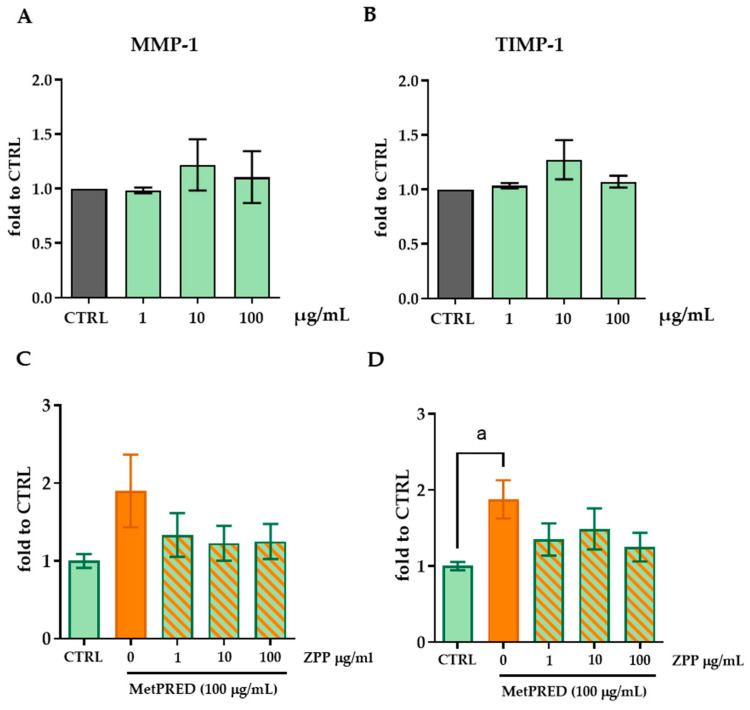
Analysis of MMP-1 (**A**) and TIMP-1 (**B**) protein levels by ELISA assay in HFF cell lysate after treatment with ZPP at the concentrations of 1, 10, and 100 µg/mL for 24 h. Data were analyzed using one-way ANOVA (MMP-1: F(3,7) = 0.340, *p* = 0.7978; TIMP-1: F(3,7) = 1.445, *p* = 0.3089) followed by Dunnett’s post hoc multiple comparison (*p* > 0.05). Dosage of MMP-1 (**C**) and TIMP-1 (**D**) protein levels in HFF cell lysate after pre-treatment for 2 h with ZPP at the concentrations of 1, 10, and 100 µg/mL then exposed to MetPRED (100 µg/mL) for 6 h. One-way ANOVA (MMP-1: F(4,14) = 1.551, *p* = 0.2450; TIMP-1: F(4,13) = 2.795, *p* = 0.0709) followed by Tukey’s post hoc multiple comparison; (a) *p* < 0.05 vs. CTRL, (n = 6). Data are expressed as mean ± SD. Control cells (CTRL) were arbitrarily set to 1.

The investigation into ZPP activity on the ECM continued by exploring the possible molecular mechanisms that underlie the specific activity of ZPP on collagen metabolism. Concerning this, we focused the analysis on two important factors involved in the regulation of the ECM and tissue remodeling essential for healthy tissue function and regeneration: the MMP-1 (Matrix Metalloproteinase-1) and TIMP-1 (Tissue Inhibitor of Metalloproteinases-1) enzymes. MMP-1 is a proteolytic enzyme whose primary function is to degrade collagen, specifically type I, II, and III collagen. MMP-1 plays a crucial role in tissue remodeling, wound healing, and the normal turnover of connective tissues. TIMP-1 is a natural inhibitor of MMPs, including MMP-1. It binds to MMPs and inhibits their enzymatic activity, thereby preventing excessive breakdown of the ECM. By balancing the activity of MMPs, TIMP-1 helps to regulate ECM integrity, tissue homeostasis, and normal cellular function. By means of ELISA assay, we measured the MMP-1 and TIMP-1 levels in HFF cells after a treatment with ZPP at 1, 10, and 100 µg/mL for 24 h. The results revealed no significant effects of ZPP in modulating the levels of MMP-1 (one-way ANOVA: F(3,7) = 0.340, *p* = 0.7978) and TIMP-1 (one-way ANOVA: F(3,7) = 1.445, *p* = 0.3089) with respect to controls (Figure 6A,B). In addition, we evaluated the impact of ZPP on the levels of those enzymes in an immunosuppressive condition induced by the corticosteroid methylprednisone (MetPRED 100 µg/mL), as previously performed [29]. Interestingly, the exposure to MetPRED increased the protein levels of both MMP-1 (one-way ANOVA: F(4,14) = 1.551, *p* = 0.2450, Figure 6C) and TIMP-1 (F(4,13) = 2.795, *p* = 0.0709, Tukey’s post hoc multiple comparison: *p* < 0.05 vs. CTRL, Figure 6D). Although not statistically significant, the pre-treatment with ZPP demonstrated a trend in reducing both the corticosteroid-induced MMP-1 and TIMP-1 levels compared to MetPRED-exposed cells (Figure 6C,D).

### 3.6. Th Effects of ZPP on Collagen Synthesis Are Mediated by the Regulation of LOX Expression

**Figure 7:**
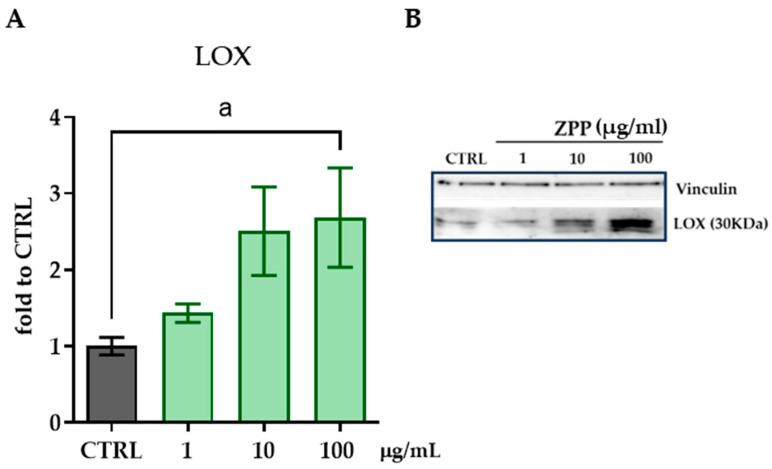
Protein levels of lysyl oxidase (LOX) enzyme measured by Western blot assay in HFF cell lysate after a 24 h-treatment with ZPP at the concentrations of 1, 10, and 100 µg/mL. (**A**) Quantification of mature protein form of LOX (30 KDa); (**B**) representative blot. Vinculin was used as loading control. Data are expressed as mean ± SD. One-way ANOVA (F(3,12) = 3.413, *p* = 0.0530) followed by Dunnett’s post hoc multiple comparison: (a) *p* < 0.05 vs. CTRL (n = 4).

Lysyl oxidase (LOX) is an enzyme that plays a crucial role in the formation and stabilization of the extracellular matrix (ECM) [40]. LOX initiates the cross-linkage of collagen and elastin by catalyzing the formation of a lysine-derived aldehyde. This cross-linking is essential for maintaining the strength, elasticity, and structural integrity of tissues such as skin, lungs, blood vessels, and bones. Considering the role of LOX, we addressed the effects of different concentrations of ZPP on the protein levels of LOX enzyme in HFFs after a 24 h treatment. Western blot analysis revealed a significant increase in LOX protein levels induced by ZPP at 10 and 100 µg/mL (one-way ANOVA: F(3,12) = 3.413, *p* = 0.0530) (Figure 7A,B).

**Figure 8:**
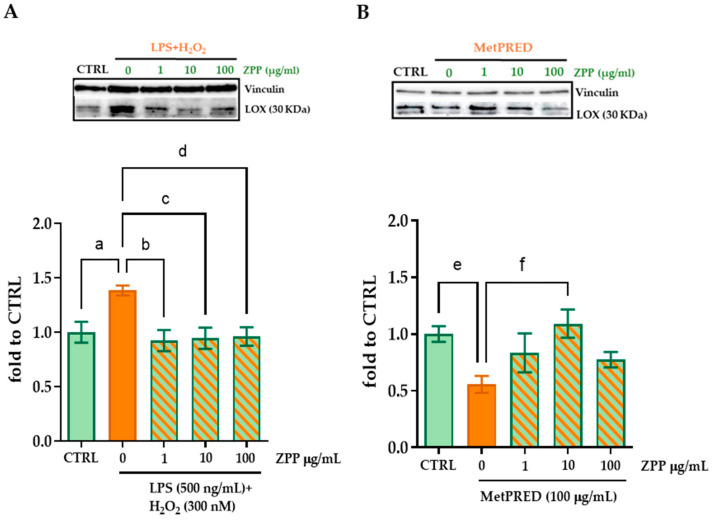
Protein levels of lysyl oxidase (LOX) enzyme measured by Western blot assay in HFF cell lysate. Cells were pre-treated for 2 h with ZPP at concentrations of 1, 10, and 100 µg/mL then stimulated with (**A**) LPS (500 ng/mL) + H_2_O_2_ (300 nM) or (**B**) MetPRED (100 µg/mL) for 6 h. Up: representative blot. Vinculin was used as loading control. Down: quantification of mature protein form of LOX (30 KDa). Data are expressed as mean ± SD. One-way ANOVA (LPS + H_2_O_2_: F(4,27) = 4.456, *p* = 0.0068; MetPRED: F(4,11) = 3.816, *p* = 0.0350) followed by Tukey’s post hoc multiple comparison: (a,e) *p* < 0.05 vs. CTRL, (b,c,d) *p* < 0.05 vs. LPS + H_2_O_2_, (f) *p* < 0.05 vs. MetPRED (n = 4).

In the same way, we attempted to determine the impact of ZPP pre-treatment on LOX levels in HFF cells under two cellular stress conditions previously optimized [29,30]: the induction of an inflammatory/oxidative stress by using the combination of stimuli LPS (500 ng/mL) + H_2_O_2_ (300 nM) or corticosteroid-induced immunosuppression (MetPRED 100 µg/mL) without affecting cell viability). We observed that the HFFs’ exposure to LPS + H_2_O_2_ induced a significant increase in LOX protein levels compared to unstimulated cells (one-way ANOVA: F(4,7) = 4.456, *p* = 0.0068,: (a) *p* < 0.05 vs. CTRL, Figure 8A), while pre-treatment with ZPP was able to inhibit this increase at all the concentrations tested (Tukey’s post hoc multiple comparison: (b,c,d) *p* < 0.05 vs. LPS + H_2_O_2_). Conversely, the MetPRED immunosuppressive stimulus induced a decrease in LOX levels compared to control cells (one-way ANOVA: F(4,11) = 3.816, *p* = 0.0350: (e) *p* <0.05 vs. CTRL, Figure 8B). Interestingly, ZPP showed effects in preventing the MetPRED-induced decrease in LOX levels, mainly at the concentration of 10 µg/mL (Tukey’s post hoc multiple comparison: (f) *p* < 0.05 vs. MetPRED) (Figure 8B). These results support the action of ZPP on collagen synthesis being plausibly due to the regulation of the expression of LOX enzyme.

### 3.7. ZPP Shows Selective Effect on Collagenase Activity and a Mild Antioxidant Capacity

In order to evaluate the ZPP inhibitory activity of the aging-related enzymes elastase, collagenase, and hyaluronidase, which are the main enzymes responsible for driving ECM degradation, a cell-free enzymatic assay was used. As observed by the IC_50_ values obtained, ZPP showed a marked selectivity in inhibiting collagenase activity, with an IC_50_ of 250 µg/mL ca., a higher inhibitory effect compared to that demonstrated on elastase and hyaluronidase activity (Table 1).

One other crucial aspect with a significant role in preventing skin aging is represented by antioxidant activity, considering that oxidative stress mediates the aging process of the skin. For this reason, by using the ORAC assay, we assessed the potential ZPP antioxidant activity. The results revealed a mild antioxidant activity of ZPP (Table 2) which may be attributed to the presence of polyphenolic compounds, particularly lignans, consistent with scientific literature [41,42].

### 3.8. ZPP Increases Elasticity and Deep Hydration and Ameliorates Skin Roughness After In Vivo Topical Application

**Figure 9:**
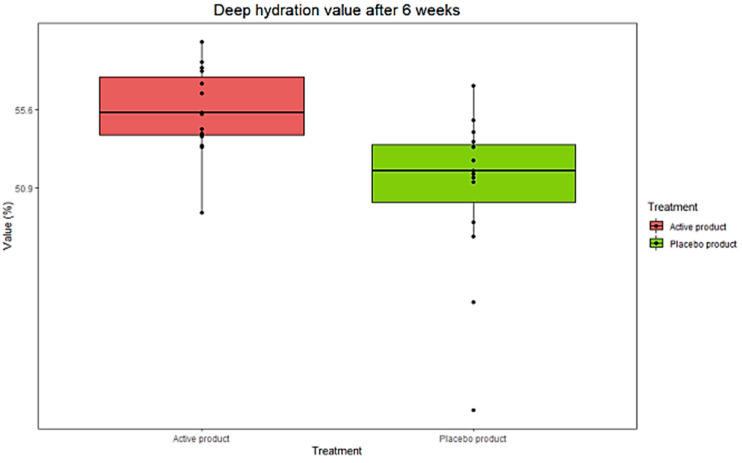
Boxplot representing distribution of collected data concerning skin deep-hydration evaluation in ZPP-treated women (active product) compared to placebo counterpart (placebo product) after 6 weeks of treatment. Data were analyzed by Friedmann’s test followed by Wilcoxon’s signed rank test for paired data, with Holm’s correction for repeated data (n = 15 for each group).

Building on the in vitro findings, we set out to evaluate the effectiveness of a topical formulation containing active ZPP by a clinical test. For this purpose, 30 female volunteers, with an average age of 52 years, were recruited and randomly assigned to two groups: one received the ZPP-containing cream, while the other was given a placebo. The treatment lasted 6 weeks, during which various parameters were assessed both periodically and at the end of the study: deep skin hydration, skin elasticity, and skin texture (Ra). After 6 weeks from the start of treatment, we observed an increase in deep hydration corresponding to +1.4% in the skin of the women who received the ZPP-containing cream compared to the placebo group (*p* < 0.05 vs. placebo) (Figure 9 and Table 3).

**Figure 10:**
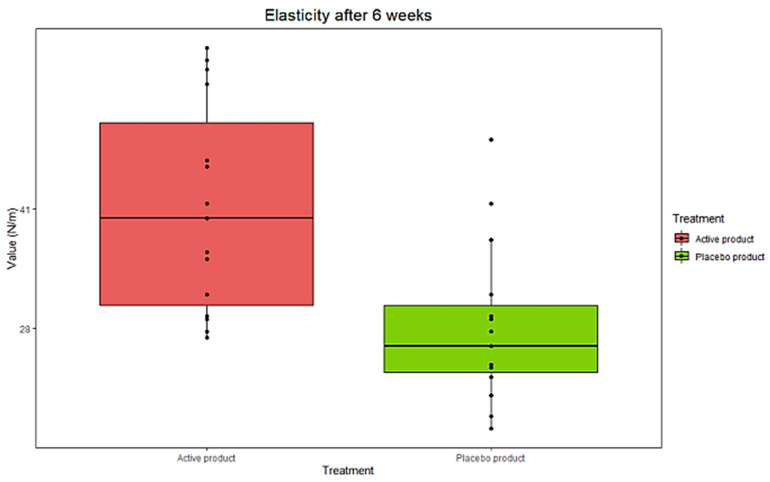
Boxplot representing distribution of collected data concerning skin elasticity evaluation in ZPP-treated women (active product) compared to placebo counterpart (placebo product) after 6 weeks of treatment. Data were analyzed by Friedmann’s test followed by Wilcoxon’s signed rank test for paired data, with Holm’s correction for repeated data (n = 15 for each group).

Regarding skin elasticity, a comparison between subjects treated with the ZPP cream and those receiving the placebo after 6 weeks showed a significant increase in skin elasticity values in the ZPP treatment group. In contrast, no significant differences were observed in the average data collected from subjects treated with the placebo (*p* < 0.05 ZPP vs. placebo) (Figure 10 and Table 4).

Concerning skin roughness, the anti-wrinkle efficacy was assessed in the décolleté area as the first area of the body to be affected by signs of aging. A rapid decrease in skin elasticity occurs above all on the neck and décolleté during aging due to their thinner skin, constant exposure to environmental factors, and frequent movement.

**Figure 11:**
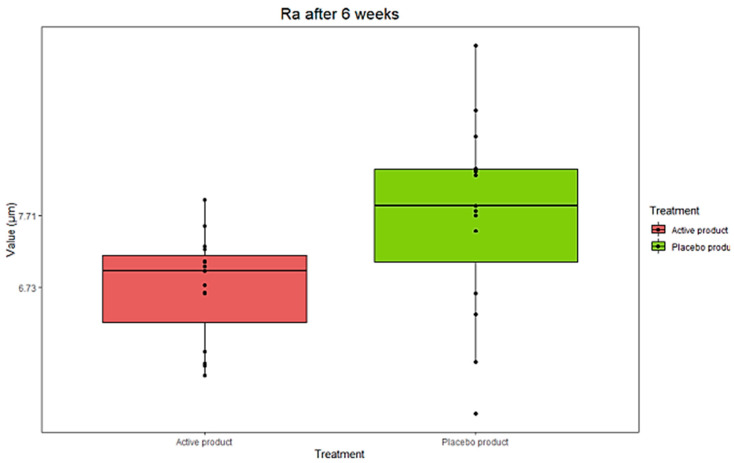
Boxplot representing distribution of data concerning skin roughness evaluation collected from the comparison between ZPP-treated group (active product) and placebo group (placebo product) after 6 weeks of treatment. Data are expressed as Ra (average value of all deviations from a straight line). Data were analyzed by Friedmann’s test followed by Wilcoxon’s signed rank test for paired data, with Holm’s correction for repeated data (n = 15 for each group).

**Figure 12:**
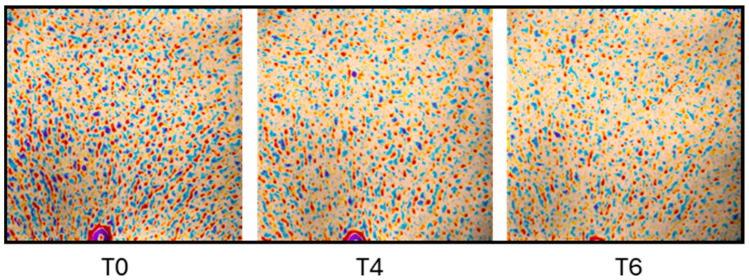
Representative images collected from the central area of the décolleté of a subject by using the Antera 3D system at different analysis time points: T0 = starting time; T4 = 4 weeks after treatment; T6 = 6 weeks after treatment. A false-color map is used to identify areas with skin roughness, where red and yellow colors represent depressed areas, and blue and purple colors represent elevated areas.

The analysis revealed a decrease in the Ra value (−20.6%) in women receiving the active ZPP cream after 6 weeks compared to the placebo counterpart (*p* < 0.1 ZPP vs. placebo) (Figure 11 and Table 5). Moreover, the skin topography showed a significant improvement in the elasticity of skin during and after the treatment with the ZPP-based cream in the evaluation of roughness (Figure 12).

Overall, the results obtained by the clinical test demonstrated the great properties of ZPP in improving the deep hydration, elasticity, and roughness of the skin.

## 4. Discussion

Effectiveness, safety, innovation, and sustainability are four key pillars of modern cosmetics. These priorities have increasingly driven the scientific community to focus on natural products, particularly medicinal plants. This study was conceptualized based on these principles and provided interesting evidence for the promising potential of a novel biotechnological *Z. piperitum* phytocomplex (ZPP) for skin health, particularly in promoting collagen synthesis, maintaining skin elasticity, and mitigating the effects of cellular stress. Previous evidence so far had reported the anti-inflammatory, antinociceptive, and anti-bacterial properties of *Z. piperitum* in different pathological contexts [5,7,12,43,44].

The findings clarified how ZPP interacts with human fibroblasts, with a focus on its ability to protect the ECM and support skin functions at the molecular level [13]. The biotechnological production of *Z. piperitum* extract from a stable cell line resulted in a peculiar phytocomplex rich in lignans (3.02% *w*/*w*) and free of alkylamides. These results confirmed that the chosen culture parameters successfully promoted the biosynthesis of target compounds, which may contribute to the overall bioactivity of ZPP and allow us to consolidate the use of plant cell culture technology in the research and production of standardized extracts for cosmetics and food supplementation, as well as offering sustainable alternative sources of plant ingredients from slow-growing plants with low environmental impact [24].

ZPP was found to be non-toxic to human fibroblasts, the main cells constituting the skin dermis, at all tested concentrations, affirming its safety for use in skin-related applications. These observations set the stage for exploring its biological effects, particularly in conditions that mimic environmental stressors such as osmotic stress. In the context of cellular stress, ZPP demonstrated a protective effect. The pre-treatment of fibroblasts with ZPP before exposure to hyperosmotic conditions reduced cell damage, with the highest concentration (100 µg/mL) significantly restoring cell viability. This suggested that ZPP may strengthen cellular resilience in stressful conditions, which is particularly relevant for maintaining skin integrity under environmental stressors like dehydration or osmotic imbalance.

The antioxidant effect and collagen synthesis stimulation of ZPP were also shown, providing promising premises for enhancing skin health [45,46,47,48]. In fact, one of the most intriguing findings was the effect of ZPP on collagen metabolism, a pivotal element in almost all aging-related skin dysregulation. While ZPP did not influence elastin production, it slightly enhanced pro-collagen I synthesis at the highest concentration (100 µg/mL). Although the increase was not statistically significant, it indicates a trend toward improved collagen production, a key factor in maintaining skin firmness and structure. This effect could be likely mediated by the upregulation of lysyl oxidase (LOX), a critical enzyme responsible for collagen cross-linking and ECM stabilization. More notably, ZPP significantly increased LOX protein levels, both in normal conditions and under stress conditions induced by pro-inflammatory stimuli (LPS + H_2_;O_2_;) and corticosteroid-induced immunosuppression.

To our knowledge, this is the first evidence of a *Z. piperitum* regulatory activity for LOX expression, suggesting that ZPP enriched in lignans may support the stabilization and maturation of collagen fibers, contributing to its potential anti-aging activity. Actually, despite a huge interest in testing new cosmetic ingredients, the specific topic of collagen metabolism is still yet to be comprehensively investigated in the field of natural products, and few herbal products have been reported to be effective at low and reliable concentrations, ≤100 μg/mL: among these, a noteworthy effect in collagen biosynthesis upregulation in fibroblasts has been demonstrated for *Ipomoea pes-caprae* (L.) R.Br. ethanolic extracts [49] and *Phyllanthus indofischeri* Bennet extracts [50]. With regard to studies which have taken LOX expression into consideration for skin aging, the most promising one was related to the combination of *Anethum graveolens* L. and *Rubus fruticosus* L. extracts [51] at 3 mg/mL that showed activity in upregulating LOX, elastin, and collagen levels.

The efficacy of ZPP, especially under both oxidative/inflammatory and immunosuppressive conditions, reinforced its potential role in protecting and maintaining the integrity of the ECM and promoting skin regeneration. This regulatory role of ZPP on LOX expression suggests it could be particularly beneficial for individuals with compromised skin elasticity or structure due to aging or prolonged corticosteroid use [13,44,47,52]. Moreover, ZPP selectively inhibited collagenase activity, further confirming its specific ability to protect collagen from enzymatic degradation. This selective inhibition is particularly relevant in aging-related skin concerns, where excessive collagenase activity leads to collagen breakdown and the loss of skin structure.

In terms of ECM degradation mechanisms, ZPP’s effects on MMP-1 and TIMP-1 offered additional insights. MMP-1, a collagenase enzyme responsible for collagen breakdown, and its natural inhibitor TIMP-1, were not significantly modulated by ZPP in basal conditions. However, under an immunosuppressive model induced by methylprednisolone, that mimicked the downregulation of fibroblast turnover and collagen synthesis typical of aged skin, both MMP-1 and TIMP-1 levels significantly increased. Although ZPP did not prevent in a statistical manner the upregulation of MMP-1 (and that of TIMP-1) induced by corticosteroids, it demonstrated a trend toward mitigating this effect, suggesting that ZPP may help to maintain ECM integrity by partially controlling MMP-1 activity under stress conditions. This finding, while not statistically significant, provides a rationale for further investigation into the regulatory role of ZPP on ECM remodeling enzymes.

ZPP further showed mild antioxidant activity, supporting its potential use as an anti-aging agent [10,12,48]. However, the relatively modest antioxidant activity (70.80 µmol TEAC/g) suggests that ZPP may need to be combined with other antioxidants for optimal protection against oxidative stress.

Finally, the in vivo results strongly support the in vitro findings. The topical application of ZPP-containing cream formulation significantly improved skin hydration, elasticity, and roughness in volunteers over a six-week period. These improvements reflect ZPP’s ability to enhance skin moisture retention and structural integrity, likely due to its effects on collagen synthesis and protection against ECM degradation. Although the measurement data are not statistically significant, the clinical observations showed significant differences in reducing wrinkles on the tested area, suggesting that a long-term treatment or higher concentrations of ZPP could most likely reinforce its effectiveness and enrich the fields of application for ZPP. These outcomes align with the in vitro findings and suggest that ZPP can improve overall skin quality, particularly in aging or dehydrated skin.

In vivo tests, coupled with cell-based insights, represent a fundamental step that should be always considered in the development of new skin anti-aging natural agents, but, so far, only a limited number of studies have been published which at the same time meet the criteria of a well-characterized extract, the investigation of the mechanisms of action in skin cells, and in vivo corroboration using low and realistic concentrations. However, in the last three years, results worthy of being cited have been obtained for different categories of plant-based products, such as for the edible plant *Vigna angularis* (Willd.) Ohwi and H. Ohash [53] (azuki beans), for the standardized extract BG100 from *Kaempferia parviflora* Wall. ex-Baker rhizomes [54], and also for marine products, such as the alga *Dunaliella salina* Teodoresco, E.C. [55]. Our study adds to the list of herbal extracts, tested both in vitro and in vivo, a new biotechnological standardized product, namely, ZPP.

### Limitations of the Study

Despite interesting findings, we are clearly aware that this study has some limitations that we do not underrate. Being a phytocomplex with a multitarget effect, already demonstrated in the In vitro tests of this work, it was very difficult to identify a unique “positive control” to be included in In vitro and in vivo tests; the aim of this first study was to explore the biological activity of the whole *Z. piperitum* phytocomplex in both multistep cell-based and in vivo models in which we were finally able to underline the biological activity of ZPP on age-related skin effects. This first exploratory study has certainly provided us with very encouraging data on the potential effect of ZPP on collagen metabolism and on the use of this phytocomplex in vivo that deserves further investigation. Moreover, it is noteworthy that, despite the In vitro data not often being supported by statistics and the need for further investigation with larger sample sizes, the ZPP efficacy in a cosmetic formulation was well demonstrated by clinical tests. In light of the results obtained in this study, especially the effect of ZPP on collagen metabolism, further research at the cellular and molecular levels are necessary to confirm and better understand the mechanisms of action of ZPP, also comparing the phytocomplex to reference cosmetic products.

## 5. Conclusions

In summary, the biotechnological *Z. piperitum* phytocomplex (ZPP) exhibits promising properties as a cosmetic ingredient, particularly for anti-aging and skin-protective formulations. Its ability to modulate collagen synthesis, enhance LOX expression, and protect cells under stress conditions, combined with its selective collagenase inhibition, suggests that ZPP has a significant potential as an ingredient in skin care products aimed at enhancing skin firmness, elasticity, and overall structural integrity, particularly in aging or stressed skin conditions. The in vivo improvements in hydration and elasticity further support its potential efficacy in skin care and protection. Future studies should explore long-term effects and optimize formulation strategies to maximize the benefits of ZPP for skin health.

## Figures and Tables

**Table 1:** ZPP inhibitory activity of collagenase, elastase and hyaluronidase enzymes measured by means of cell-free enzymatic essays. Data are expressed as IC_50_ (µg/mL), mean ± SD.

	Collagenase	Elastase	Hyaluronidase
IC_50_ (µg/mL)	253.59 ± 29.01	1036.41 ± 41.40	>1500

**Table 2:** Antioxidant activity of *Z. piperitum* phytocomplex measured by ORAC assay. Data are expressed as mean ± SD referred to equivalent concentration of Trolox.

	μmol TEAC/g
ZPP	70.80 ± 8.05

**Table 3:** Data obtained on skin deep-hydration measurement at starting time (T0) and after 6 weeks of treatment (Tf), with ZPP-containing cream or placebo. Variation normalized to the mean of T0 values is in brackets. Data are expressed as mean ± standard deviation (SD) and were analyzed by Friedmann’s test followed by Wilcoxon’s signed rank test for paired data, with Holm’s correction for repeated data (n = 15 for each group). *: *p* < 0.05 ZPP cream vs. placebo cream.

	T0 ZPP Cream (%)	Tf Placebo Cream (%)	Variation Tf–T0 (%)	Variation Active–Placebo (*p-*Value)
ZPP cream	52.8 ± 2.5	55.6 ± 2.6	+2.8%	+1.4% (+2.8%) *(p* < 0.05 *)
Placebo cream	49.5 ± 6.8	50.9 ± 4.9	+1.4%	

**Table 4:** Comparison values of skin elasticity measured at starting time (T0) and after 6 weeks of treatment (Tf) with the ZPP-containing cream or placebo. Variation normalized to the mean of T0 values is in brackets. Data are expressed as mean ± standard deviation (SD) and were analyzed by Friedmann’s test followed by Wilcoxon’s signed rank test for paired data, with Holm’s correction for repeated data (n = 15 for each group). *: *p* < 0.05 ZPP cream vs. placebo cream.

	T0 ZPP Cream (N/m)	Tf Placebo Cream (N/m)	Variation Tf–T0 (N/m)	Variation Active–Placebo (*p-*Value)
ZPP cream	23 ± 9	41 ± 12	+18 N/m	+9 N/m (+43.2%) *(p* = 0.02 *)
Placebo cream	19 ± 8	28 ± 9	+9 N/m	

**Table 5:** Comparison values of skin roughness measured at starting time (T0) and after 6 weeks of treatment (Tf) with the ZPP-containing cream or placebo. Variation normalized to the mean of T0 values is in brackets. Data are expressed as mean ± standard deviation (SD) and were analyzed by Friedmann’s test followed by Wilcoxon’s signed rank test for paired data, with Holm’s correction for repeated data (n = 15 for each group). *: *p* < 0.1 ZPP cream vs. placebo cream.

	T0 ZPP Cream (μm)	Tf Placebo Cream (μm)	Variation Tf–T0 (μm)	Variation Active–Placebo (*p-*Value)
ZPP cream	8.2 ± 1.5	6.7*±* 0.7	−1.5	−1.3 µm (−20.6%) *(p* = 0.08 *)
Placebo cream	7.9 ± 1.3	7.7*±* 1.3	−0.2	

## Data Availability

The authors do not have permission to share data.

## Supplementary Materials

The supporting information can be downloaded at: https://www.mdpi.com/article/10.3390/pharmaceutics17010138/s1.
