# Supplementary material for: Biotechnological Phytocomplex of Zanthoxylum piperitum (L.) DC. Enhances Collagen Biosynthesis In Vitro and Improves Skin Elasticity In Vivo
Source: Pharmaceutics. 2025 Jan 20;17(1):138. doi: 10.3390/pharmaceutics17010138 (PMC11768096; doi:10.3390/pharmaceutics17010138)
Supplement: Supplementary file 1 [file pharmaceutics-17-00138-s001.zip › pharmaceutics-3379956-supplementary.pdf]

Supplementary information

Material and methods

1. Manufacture of cosmetic formulation

The formulation is an O/W (oil in water) emulsion and consists of two phases. Oil phase and water phase. The water phase contains water, glycerin and gelling polymers. The oil phase contains different types of oils, an antioxidant and the emulsifier. Both phases were heated up to 75 °C and then the oil phase was placed in the water phase under a mixer for a few minutes. Then the temperature was lowered to 25 °C under a stirrer. The formulation was completed with preservatives, citric acid and with ZP (*Zanthoxylum piperitum* Callus Lysate) dispersed in 2% w/w vegetable glycerol. The final ZP content in the cream was 0.07% w/w. The ingredients in the formulation are denominated according to INCI (International Nomenclature of Cosmetic Ingredients) in the cosmetic industry and are listed as follow in a descending order according to ingredients concentration in the cosmetic formulation: Aqua, Glycerin, Ethylhexyl Palmitate, Coco-Caprylate, Cetearyl Olivate, Sorbitan Olivate, Caprylic/Capric Tryglyceride, Butyrospermum Parkii Butter, Cetearyl Alcohol, Cetyl Palmitate, Dicaprylyl Ether, Sorbitan Palmitate, Ammonium Acryloyldimethyltaurate/VP Copolymer, Hydroxyacetophenone, 1,2-Hexanediol, Capryl Glycol, Tocopherol, Xanthan Gum, Polyhydroxystearic Acid, Sodium Gluconate, *Zanthoxylum piperitum* Callus Lysate, Lecithin, Citric Acid, Ascorbyl Palmitate. The placebo cream contained the same ingredients, except for ZP.

2. Safety assessment of cosmetic formulation

ZPP dispersed in vegetable glycerin (INCI: Glycerin, *Zanthoxylum Piperitum* Callus Lysate) is the only new ingredient used in the cosmetic formula for *in vivo* test. All other ingredients used in the formula are widely used in cosmetics. The ingredients widely used in cosmetics have already been tested regarding their safety. To show the safety and tolerability of the new ingredient, ZPP was tested by a patch test occlusive under dermatological control (Eurochem s.r.l., RAP 43688). The test was carried out on 20 healthy volunteers of both sexes, selected after application of inclusion/non-inclusion criteria. The ZPP dispersed in vegetable glycerin was posed into aluminum Finn Chambers and applied to the skin of the back. The cosmetic ingredient was left in contact with the skin surface for 48 hours. Removal of the Finn Chamber and cleaning of the skin area from residual cosmetic product was carried out by the experimenter. The visual assessment of skin irritation was made 15 minutes and 48 hours after removal of Finn Chambers (48 and 96 hours after product application), according to the scoring scale. The ZPP dispersed in vegetable glycerin, applied, AS IT IS under occlusive condition on the healthy skin of 20 volunteers, resulted in a mean index of irritation of:

IRRITATION INDEX	TIME
0.00	15 minutes after patch removal (48 hours after product application)
0.00	48 hours after patch removal (96 hours after product application)

According to evaluation scale used, the product can be classified as not irritating if applied to intact human skin.
